# The genome sequence of *Russula nigricans* Fr., 1838 (Russulales: Russulaceae)

**DOI:** 10.12688/wellcomeopenres.28091.1

**Published:** 2026-10-09

**Authors:** Yvonne Mynett, Anna Bazzicalupo, Alex Dombrowski, Ben Blades, Ester Gaya, Clare Blencowe, Royal Botanic Gardens Kew Genome Acquisition Lab, Darwin Tree of Life Barcoding collective, Wellcome Sanger Institute Tree of Life Management, Samples and Laboratory team, Wellcome Sanger Institute Scientific Operations: Sequencing Operations, Wellcome Sanger Institute Tree of Life Core Informatics team, Tree of Life Core Informatics collective, Darwin Tree of Life Consortium

**Affiliations:** 1 British Mycological Society, Wrexham, UK; 2 Royal Botanic Gardens Kew, Richmond, Surrey, England, UK

**Keywords:** Russula nigricans, Blackening Brittlegill, genome sequence, chromosomal, Russulales

## Abstract

We present a genome assembly of *Russula nigricans* (Blackening Brittlegill; Basidiomycota; Agaricomycetes; Russulales; Russulaceae). The assembly consists of two haplotypes with total lengths of 44.21 megabases and 45.01 megabases. Haplotypes 1 and 2 each contain 11 chromosomal pseudomolecules; these account for 99.88% of haplotype 1 and all of haplotype 2. The mitochondrial genome has been assembled, with a length of 54.23 kilobases. Gene annotation of this assembly on Ensembl identified 13,918 protein-coding genes. This assembly was generated as part of the Darwin Tree of Life project, which produces reference genomes for eukaryotic species found in Britain and Ireland.

## Species taxonomy

Eukaryota; Opisthokonta; Fungi; Dikarya; Basidiomycota; Agaricomycotina; Agaricomycetes; Agaricomycetes incertae sedis; Russulales; Russulaceae; *Russula*; *Russula nigricans* Fr., 1838 (NCBI:txid152964)

## Background

*Russula nigricans* (Blackening Brittlegill) is a common mushroom-forming species in the ectomycorrhizal fungal genus *Russula*. *Russula* is a diverse, species-rich genus with 700–900 accepted species names ( Kirk *et al.* , 2008) and more than 1,000 operational taxonomic units (OTUs) identified using the ITS DNA barcode ( Looney *et al.*, 2016). *Russula nigricans* belongs to subgenus *Compactae* ( Buyck *et al.*, 2018; De Lange *et al.* , 2023). In the past, the species was synonymised with *Russula adusta*, but was subsequently separated taxonomically ( Melot, 2021) and confirmed phylogenetically as a distinct species ( De Lange *et al.* , 2023).

*Russula nigricans* produces a large fruit body that reddens and later blackens when bruised. The gills are thick, brittle and widely spaced. It has a mild but unpleasant taste. Spores measure 6.5–8 × 6–7 µm and are verrucose with connecting lines ( Læssøe & Petersen, 2019).

Throughout the UK, it grows on soil under both broadleaved and coniferous trees and is not associated with any specific tree host. Decaying fruit bodies of *Russula nigricans* often host the Silky Piggyback ( *Asterophora parasitica*) and Powdery Piggyback ( *Asterophora lycoperdoides*) fungi. With reference genomes for both *Asterophora* species already available (BioProject PRJNA417766), publication of the *Russula nigricans* genome will facilitate genomic investigation of these host–parasite relationships.

We sequenced the genome of *Russula nigricans* as part of the Darwin Tree of Life Project, which aims to generate high-quality reference genomes for all named eukaryotic species in Britain and Ireland to support research, conservation and the sustainable use of biodiversity. The sequenced specimen was collected from Cragside, Northumberland, England, UK (Figure 1).

**Figure 1.**
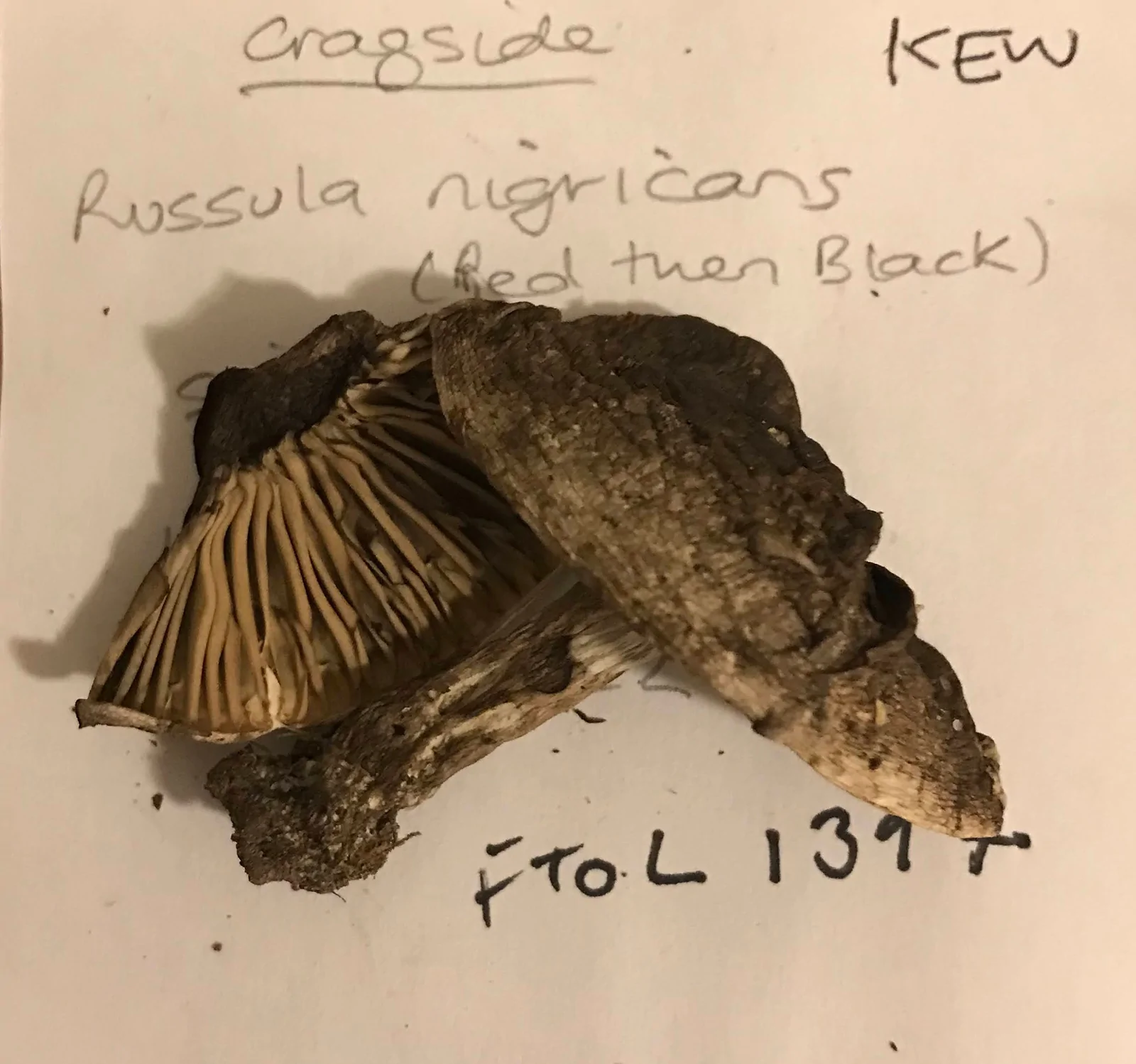
*R. nigricans* specimen (gfRusNigr1) used for genome sequencing.

## Methods

### Sample acquisition and DNA barcoding

A specimen of *R. nigricans* (specimen ID KDTOL00866, ToLID gfRusNigr1; Figure 1) was used for genome sequencing. It was collected from Cragside, Northumberland, England, UK (latitude 55.308, longitude −1.891) on 2022-10-01. The specimen was collected and identified by Yvonne Mynett (British Mycological Society). The gfRusNigr1 specimen was also used for Hi-C and RNA sequencing.

The initial identification was verified by DNA barcoding according to the framework developed by Twyford *et al*. (2024). DNA was extracted from the dried fruiting body with the use of a QIAGEN DNA extraction kit. The internal transcribed spacer (ITS) region was amplified by PCR, and amplicons were sequenced at the Natural History Museum, and compared to sequences available on Genbank and UNITE. After verification, the samples were shipped on dry ice to the Wellcome Sanger Institute (WSI) for whole genome sequencing.

### Nucleic acid extraction

Protocols for high molecular weight (HMW) DNA extraction developed at the Wellcome Sanger Institute (WSI) Tree of Life Core Laboratory are available on protocols.io ( Howard *et al.*, 2025). The gfRusNigr1 sample was weighed and triaged to determine the appropriate extraction protocol.

For HMW DNA extraction, 50.0 mg of mycelium tissue was homogenised by cryogenic disruption using the Covaris cryoPREP ^®^ Automated Dry Pulverizer. DNA was extracted using the Automated Plant MagAttract v4 protocol. We used centrifuge-mediated fragmentation to produce DNA fragments in the 8–10 kb range, following the Covaris g-TUBE protocol for ultra-low input (ULI). Sheared DNA was purified by automated SPRI (solid-phase reversible immobilisation), using AMPure PB beads (Pacific Biosciences) and the Thermo Fisher KingFisher™ Apex to eliminate shorter fragments and concentrate the DNA.

The concentration of the sheared and purified DNA was assessed using a NanoDrop spectrophotometer and Qubit Fluorometer using the Qubit dsDNA High Sensitivity Assay kit. Fragment size distribution was evaluated by running the sample on the FemtoPulse system. For this sample, final post-shearing DNA measurements included a Qubit concentration of 0.768 ng/μL, and an average fragment size of 7.3 kb.

RNA was extracted from mycelium tissue of gfRusNigr1 in the Tree of Life Laboratory at the WSI using the RNA Extraction: Automated MagMax™ *mir*Vana protocol. The RNA concentration was assessed using a NanoDrop spectrophotometer and a Qubit Fluorometer using the Qubit RNA Broad-Range Assay kit. RNA integrity was assessed using the Agilent RNA 6000 Pico Kit and Eukaryotic Total RNA assay.

### PacBio HiFi library preparation and sequencing

Library preparation and sequencing were performed at the WSI Scientific Operations core. Prior to library preparation, the DNA was fragmented to ~10 kb. Ultra-low-input (ULI) libraries were prepared using the PacBio SMRTbell® Express Template Prep Kit 2.0 and gDNA Sample Amplification Kit. Samples were normalised to 20 ng DNA. Single-strand overhang removal, DNA damage repair, and end-repair/A-tailing were performed according to the manufacturer’s instructions, followed by adapter ligation. A 0.85× pre-PCR clean-up was carried out with Promega ProNex beads.

The DNA was evenly divided into two aliquots for dual PCR (reactions A and B), both following the manufacturer’s protocol. A 0.85× post-PCR clean-up was performed with ProNex beads. DNA concentration was measured using a Qubit Fluorometer v4.0 (Thermo Fisher Scientific) with the Qubit HS Assay Kit, and fragment size was assessed on an Agilent Femto Pulse Automated Pulsed Field CE Instrument (Agilent Technologies) using the gDNA 55 kb BAC analysis kit. PCR reactions A and B were then pooled, ensuring a total mass of ≥500 ng in 47.4 μl.

The pooled sample underwent another round of DNA damage repair, end-repair/A-tailing, and hairpin adapter ligation. A 1× clean-up was performed with ProNex beads, followed by DNA quantification using the Qubit and fragment size analysis using the Agilent Femto Pulse. Size selection was performed on the Sage Sciences PippinHT system, with target fragment size determined by Femto Pulse analysis (typically 4–9 kb). Size-selected libraries were cleaned with 1.0× ProNex beads and normalised to 2 nM before sequencing.

The sample was sequenced on a Revio instrument (Pacific Biosciences, California, USA). Prepared libraries selected for multiplexing were pooled based on genome size, library molarity, and the intended plex level. Primers were annealed and polymerases were bound to generate circularised complexes, following the manufacturer’s instructions. Complexes were purified using SMRTbell beads, diluted to the Revio loading concentration, and spiked with a Revio sequencing internal control. The pooled libraries were sequenced on a Revio 25M Plex SMRT cell in a 4-plex run.

SMRT Link software (Pacific Biosciences), a web-based workflow manager, was used to configure and monitor the run and to carry out primary and secondary data analysis.

### Hi-C

***Sample preparation and crosslinking***

Hi-C data were generated from the mycelium tissue of gfRusNigr1 using the Arima-HiC v2 kit (Arima Genomics). Tissue was finely ground using the Covaris cryoPREP Dry Pulverizer (Covaris), and then subjected to nuclei isolation. Nuclei were isolated using a modified protocol based on the Qiagen QProteome Cell Compartment Kit (Qiagen), in which only the Lysis and CE2 buffers were used, with QIAshredder spin columns. After isolation, nuclei were fixed using formaldehyde to a final concentration of 2% to crosslink the DNA. The crosslinked DNA was then digested and biotinylated according to the manufacturer’s instructions. A clean-up step was performed with SPRIselect beads before library preparation. DNA concentration was quantified using the Qubit Fluorometer v4.0 (Thermo Fisher Scientific) and the Qubit HS Assay Kit, following the manufacturer’s instructions.

***Hi-C library preparation and sequencing***

Biotinylated DNA constructs were fragmented using a Covaris E220 sonicator and size-selected to 400–600 bp using SPRISelect beads. DNA was enriched with Arima-HiC v2 kit Enrichment beads. End repair, A-tailing, and adapter ligation were carried out with the NEBNext Ultra II DNA Library Prep Kit (New England Biolabs), following a modified protocol where library preparation occurs while DNA remains bound to the Enrichment beads. Library amplification was performed using KAPA HiFi HotStart mix and a custom Unique Dual Index (UDI) barcode set (Integrated DNA Technologies). Depending on sample concentration and biotinylation percentage determined at the crosslinking stage, libraries were amplified with 10–16 PCR cycles. Post-PCR clean-up was performed with SPRISelect beads. Libraries were quantified using the AccuClear Ultra High Sensitivity dsDNA Standards Assay Kit (Biotium) and a FLUOstar Omega plate reader (BMG Labtech).

Prior to sequencing, libraries were normalised to 10 ng/μL. Normalised libraries were quantified again to create equimolar and/or weighted 2.8 nM pools. Pool concentrations were checked using the Agilent 4200 TapeStation (Agilent) with High Sensitivity D500 reagents before sequencing. Sequencing was performed using paired-end 150 bp reads on the Illumina NovaSeq X.

### RNA library preparation and sequencing

Libraries were prepared using the NEBNext® Ultra II Directional RNA Library Prep Kit for Illumina (New England Biolabs), following the manufacturer’s instructions. Poly(A) mRNA was isolated from 100 ng of total RNA using oligo(dT) beads, converted to cDNA, and uniquely indexed; 14 PCR cycles were performed. Libraries were prepared with an intended average fragment size of 100–300 bp. Libraries were quantified, normalised, and pooled equimolarly to a final concentration of 2.8 nM before dilution to 150 pM for loading. Sequencing was carried out on the Illumina NovaSeq 6000; Illumina NovaSeq X, generating paired-end reads.

### Genome assembly

Prior to assembly of the PacBio HiFi reads, a database of *k*-mer counts ( *k* = 31) was generated from the filtered reads using FastK. GenomeScope (version 2.1.0) ( Ranallo-Benavidez *et al.*, 2020) was used to analyse the *k*-mer frequency distribution, providing fitted point estimates of genome size, heterozygosity and repeat content.

The HiFi reads were assembled using Hifiasm in Hi-C phasing mode ( Cheng *et al.*, 2021, 2022), producing two haplotypes. Hi-C reads ( Rao *et al.* , 2014) were mapped to the primary contigs using bwa-mem2 ( Vasimuddin *et al.*, 2019). Contigs were further scaffolded with Hi-C data in YaHS ( Zhou *et al.*, 2023), using the --break option for handling potential misassemblies. The scaffolded assemblies were evaluated using Gfastats ( Formenti *et al.* , 2022), BUSCO ( Manni *et al.*, 2021) and MerquryFK ( Rhie *et al.*, 2020). The mitochondrial genome was assembled using MitoHiFi ( Uliano-Silva *et al.* , 2023) and OATK ( Zhou *et al.*, 2025). The comparator for the mitochondrial assembly was the mitochondrial genome of *Russula vinacea* (NC_088767.1).

### Assembly curation

The assembly was decontaminated using the Assembly Screen for Cobionts and Contaminants ( ASCC) pipeline. TreeVal was used to generate the flat files and maps for use in curation. Manual curation was conducted primarily in PretextView and HiGlass ( Kerpedjiev *et al.* , 2018). Scaffolds were visually inspected and corrected as described by Howe *et al*. (2021). Manual corrections included 23 breaks and 24 joins. This reduced the scaffold count by 50.0% and reduced the total assembly length by 1.2%. The curation process is described at sanger-tol/curation-resources. PretextSnapshot was used to generate a Hi-C contact map of the final assembly.

### Assembly quality assessment

The MerquryFK tool ( Rhie *et al.*, 2020) was run in a Singularity container ( Kurtzer *et al.*, 2017) to evaluate *k*-mer completeness and assembly quality for both haplotypes using the *k*-mer database ( *k* = 31) computed prior to genome assembly. The analysis outputs included assembly QV scores and completeness statistics.

The genome was analysed using the BlobToolKit pipeline, a Nextflow implementation of the earlier Snakemake version ( Challis *et al.*, 2020). PacBio reads were aligned to the assembly using minimap2 ( Li, 2018) and processed with SAMtools ( Danecek *et al.* , 2021) to generate coverage tracks. The pipeline runs BUSCO ( Manni *et al.*, 2021) using lineages identified from the NCBI Taxonomy ( Schoch *et al.*, 2020). For the three basal BUSCO lineages, eukaryota, bacteria and archaea, BUSCO genes are aligned to the UniProt Reference Proteomes database ( Bateman *et al.* , 2023) using DIAMOND BLASTp ( Buchfink *et al.*, 2021). The genome assembly is divided into chunks based on the density of BUSCO genes from the closest taxonomic lineage, and each chunk is aligned to the UniProt Reference Proteomes database using DIAMOND BLASTx. Sequences without DIAMOND BLASTx hits are extracted using seqtk and aligned to the NT database using BLASTn ( Altschul *et al.*, 1990). The outputs are consolidated into a BlobDir for visualisation in BlobToolKit. The BlobToolKit pipeline was developed using nf-core tooling ( Ewels *et al.* , 2020) and MultiQC ( Ewels *et al.*, 2016), with containerisation through Docker ( Merkel, 2014) and Singularity ( Kurtzer *et al.*, 2017).

## Genome sequence report

### Sequence data

The genome of a specimen of *R. nigricans* was sequenced using Pacific Biosciences single-molecule HiFi long reads, generating 16.02 Gb (gigabases) from 1.72 million reads, which were used to assemble the genome. GenomeScope analysis using a *p* = 2 model gave a *k*-mer-based haploid genome size estimate of 46.34 Mb, with heterozygosity of 0.72% and repeat content of 28.94%; the full-model fit was 92.55% (Figure 2). This estimate guided expectations for the assembly. The sequencing data provided approximately 331× coverage. Hi-C sequencing produced 97.01 Gb from 321.23 million read pairs, which were used to scaffold the assembly. RNA sequencing data were also generated and are available in public sequence repositories. Table 1 summarises the specimen and sequencing details.

**Figure 2.**
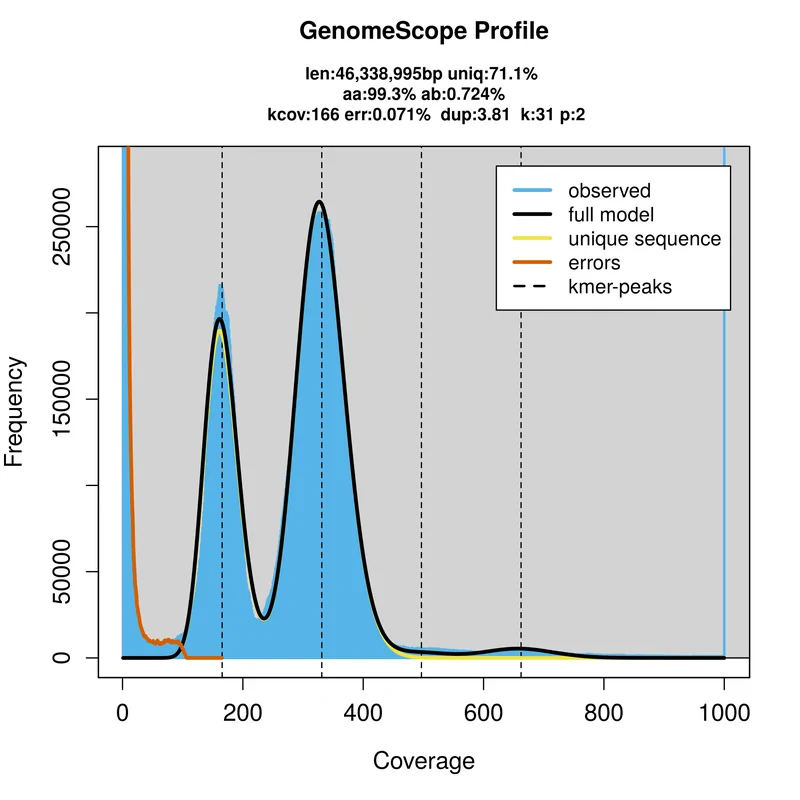
GenomeScope model fitted to the *k*-mer frequency distribution. The plot shows the observed and modelled *k*-mer spectra used to estimate genome size, heterozygosity and repeat content from unassembled sequencing reads.

**Table 1.** Specimen and sequencing data for *R. nigricans* (BioProject PRJEB77549)

**Platform**	**PacBio HiFi**	**Hi-C**	**RNA-seq**
**ToLID**	gfRusNigr1	gfRusNigr1	gfRusNigr1
**Specimen ID**	KDTOL00866	KDTOL00866	KDTOL00866
**BioSample (source individual)**	SAMEA113940631	SAMEA113940631	SAMEA113940631
**BioSample (tissue)**	SAMEA113940652	SAMEA113940652	SAMEA113940652
**Tissue**	mycelium	mycelium	mycelium
**Instrument**	Revio (1 run)	Illumina NovaSeq X	Illumina NovaSeq 6000; Illumina NovaSeq X
**Library ID & run accession(s)**	DTOL13812141: ERR13362671	ERR13363453	ERR13363452; ERR15140896
**Read count total**	1.72 million reads	321.23 million read pairs	102.86 million read pairs
**Base count total**	16.02 Gb	97.01 Gb	31.06 Gb

### Assembly statistics

The genome was assembled into two haplotypes using Hi-C phasing. Both the haplotype 1 and haplotype 2 assemblies were curated to chromosome level. The haplotype 1 assembly, which is proposed as the reference assembly for this species, has a total length of 44.21 Mb in 13 scaffolds (excluding organelle sequences), with 24 gaps, and its scaffold N50 is 4.17 Mb (Table 2). The scaffold auN for the haplotype 1 assembly is 4.15 Mb.

**Table 2.** Genome assembly statistics for *R. nigricans*

**Genome assembly**	**Haplotype 1**	**Haplotype 2**
**Assembly name**	gfRusNigr1.hap1.1	gfRusNigr1.hap2.1
**Assembly accession**	GCA_964656295.1	GCA_964656355.1
**Assembly level**	chromosome	chromosome
**Span (Mb)**	44.21	45.01
**Number of chromosomes**	11	11
**Number of contigs**	37	31
**Contig N50 (Mb)**	3.31	3.2
**Number of scaffolds**	13	11
**Scaffold N50 (Mb)**	4.17	4.24
**Longest scaffold length (Mb)**	5.4	5.74
**Organelles**	Mitochondrial genome: 54.23 kb	-

99.88% of the haplotype 1 assembly sequence was assigned to 11 chromosome-level scaffolds. These chromosome-level scaffolds, confirmed by Hi-C data, are named according to size (Figure 3; Table 3).

The mitochondrial genome was also assembled (length 54.23 kb, OZ209868.1). This sequence is included as a contig in the multifasta file of the genome submission and as a standalone record.

**Figure 3.**
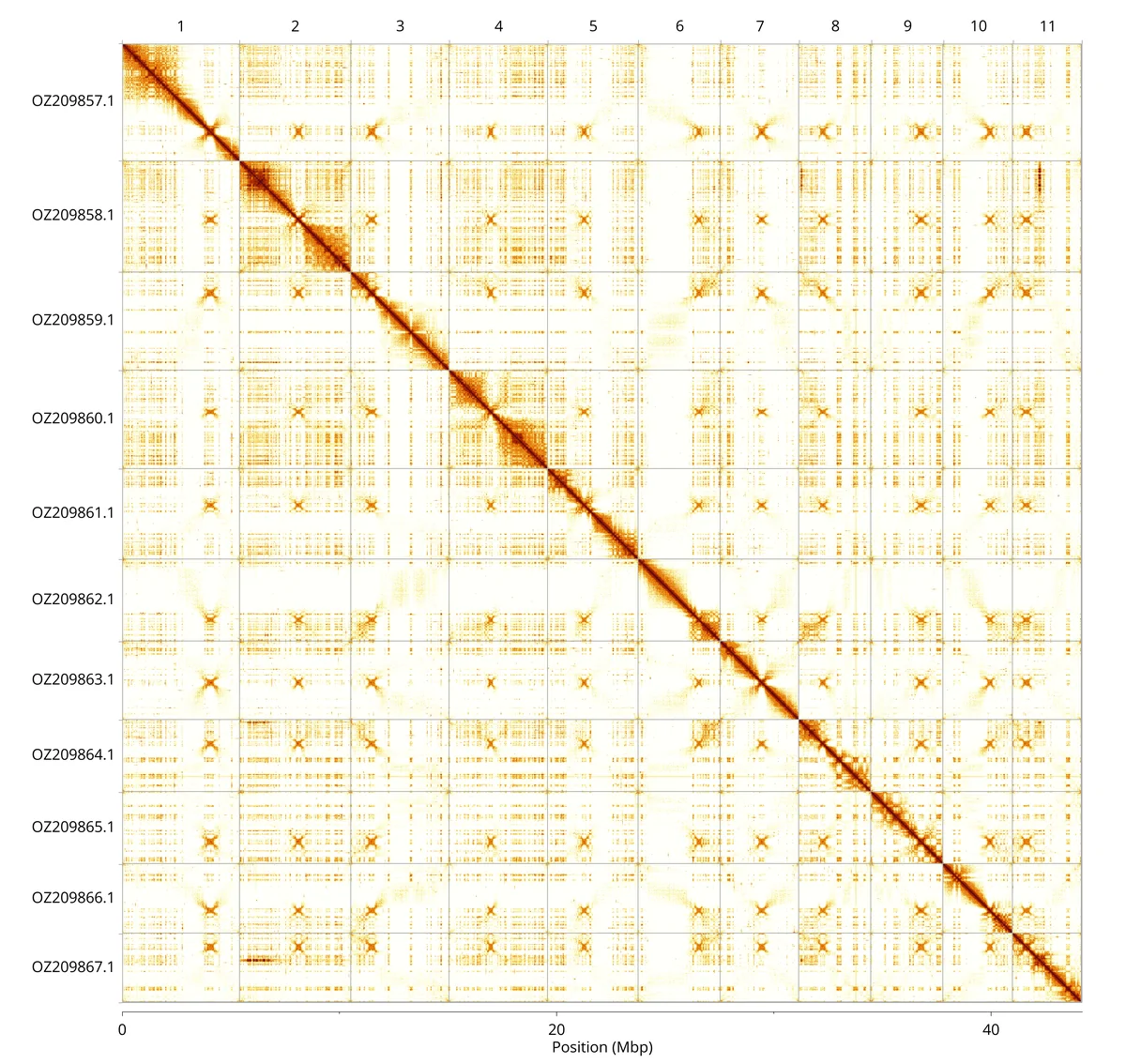
Hi-C contact map of the *R. nigricans* genome assembly. Assembled chromosomes are shown in order of size and labelled along the axes, with a megabase scale shown below. The plot was generated using PretextSnapshot.

**Table 3.** Chromosomal pseudomolecules in the haplotype 1 genome assembly of *R. nigricans* gfRusNigr1 (haplotype 2 also at chromosome level)

**INSDC accession**	**Molecule**	**Length (Mb)**	**GC%**
OZ209857.1	1	5.40	51
OZ209858.1	2	5.12	50.50
OZ209859.1	3	4.53	51.50
OZ209860.1	4	4.53	50.50
OZ209861.1	5	4.17	51
OZ209862.1	6	3.79	51.50
OZ209863.1	7	3.61	51.50
OZ209864.1	8	3.32	51
OZ209865.1	9	3.31	51
OZ209866.1	10	3.21	51
OZ209867.1	11	3.17	51

### Assembly quality metrics

For haplotype 1, the estimated QV is 64.7, and for haplotype 2, 65.7. When the two haplotypes are combined, the assembly achieves an estimated QV of 65.2. The *k*-mer completeness is 83.88% for haplotype 1, 83.96% for haplotype 2, and 99.43% for the combined haplotypes (Figure 4).

**Figure 4.**
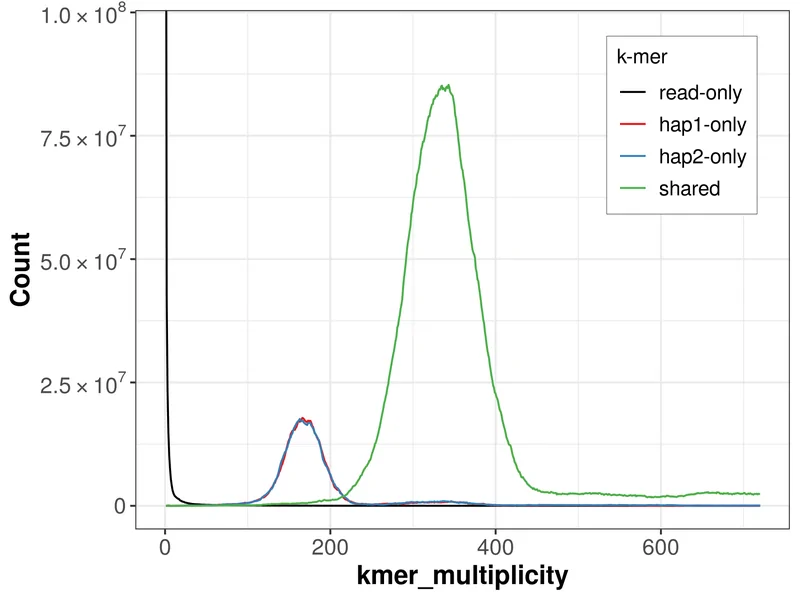
Evaluation of *k*-mer completeness using MerquryFK. This plot illustrates the recovery of *k*‐mers from the original read data in the final assemblies. The horizontal axis represents *k*‐mer multiplicity, and the vertical axis shows the number of *k*‐mers. The black curve represents *k*‐mers that appear in the reads but are not assembled. The green curve corresponds to *k*‐mers shared by both haplotypes, and the red and blue curves show *k*‐mers found only in one of the haplotypes.

BUSCO analysis using the agaricomycetes_odb10 reference set ( *n* = 2,898) identified 92.8% of the expected gene set (single = 92.3%, duplicated = 0.5%) for haplotype 1. The snail plot in Figure 5 summarises the scaffold length distribution and other assembly statistics for haplotype 1. The blob plot in Figure 6 shows the distribution of scaffolds by GC proportion and coverage for haplotype 1.

**Figure 5.**
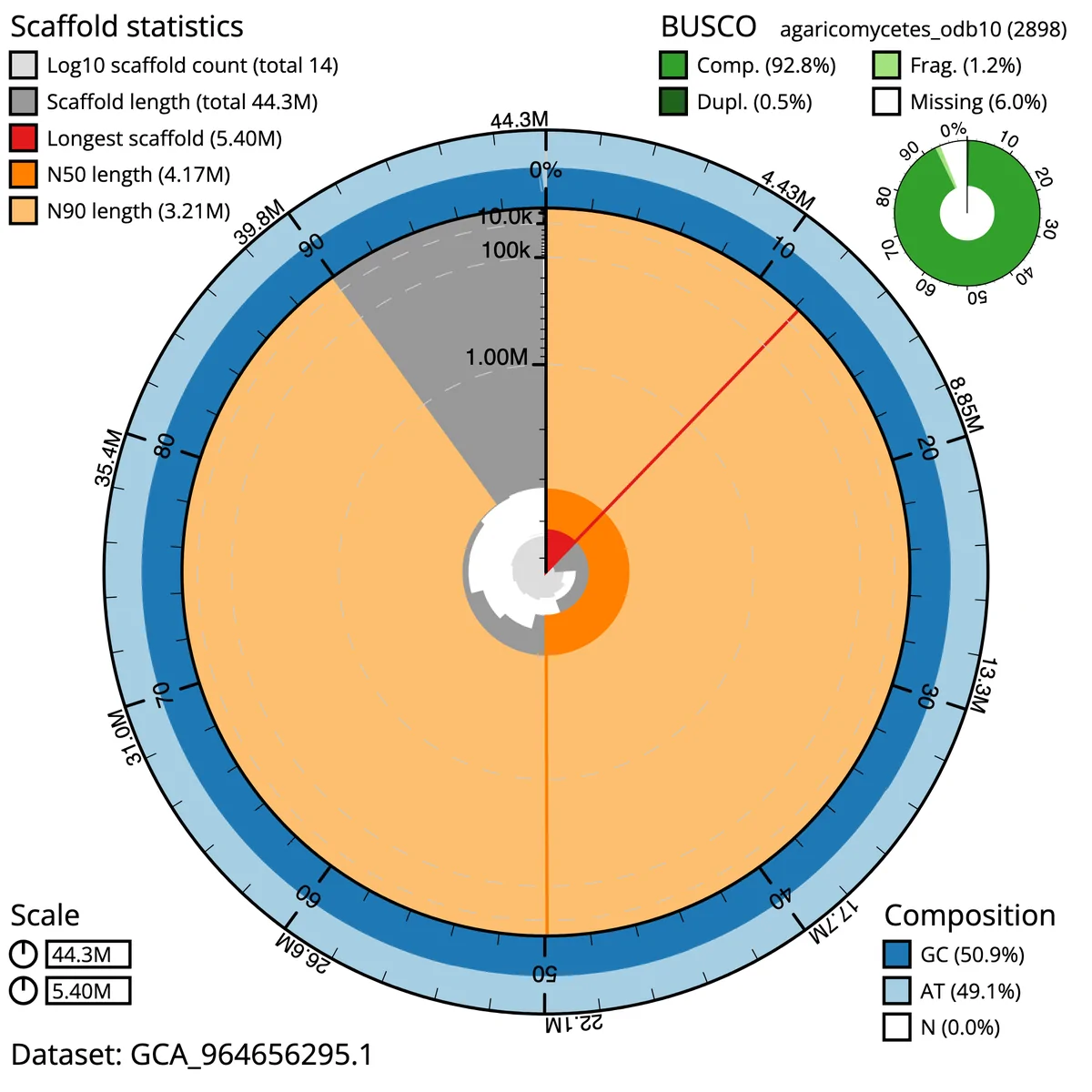
Assembly metrics for gfRusNigr1.hap1.1. The BlobToolKit snail plot provides an overview of assembly metrics and BUSCO gene completeness. The circumference represents the length of the whole genome sequence, and the main plot is divided into 1,000 bins around the circumference. The outermost blue tracks display the distribution of GC, AT, and N percentages across the bins. Scaffolds are arranged clockwise from longest to shortest and are depicted in dark grey. The longest scaffold is indicated by the red arc, and the deeper orange and pale orange arcs represent the N50 and N90 lengths. A light grey spiral at the centre shows the cumulative scaffold count on a logarithmic scale. A summary of complete, fragmented, duplicated, and missing BUSCO genes in the agaricomycetes_odb10 set is presented at the top right. An interactive version of this figure can be accessed on the BlobToolKit viewer.

**Figure 6.**
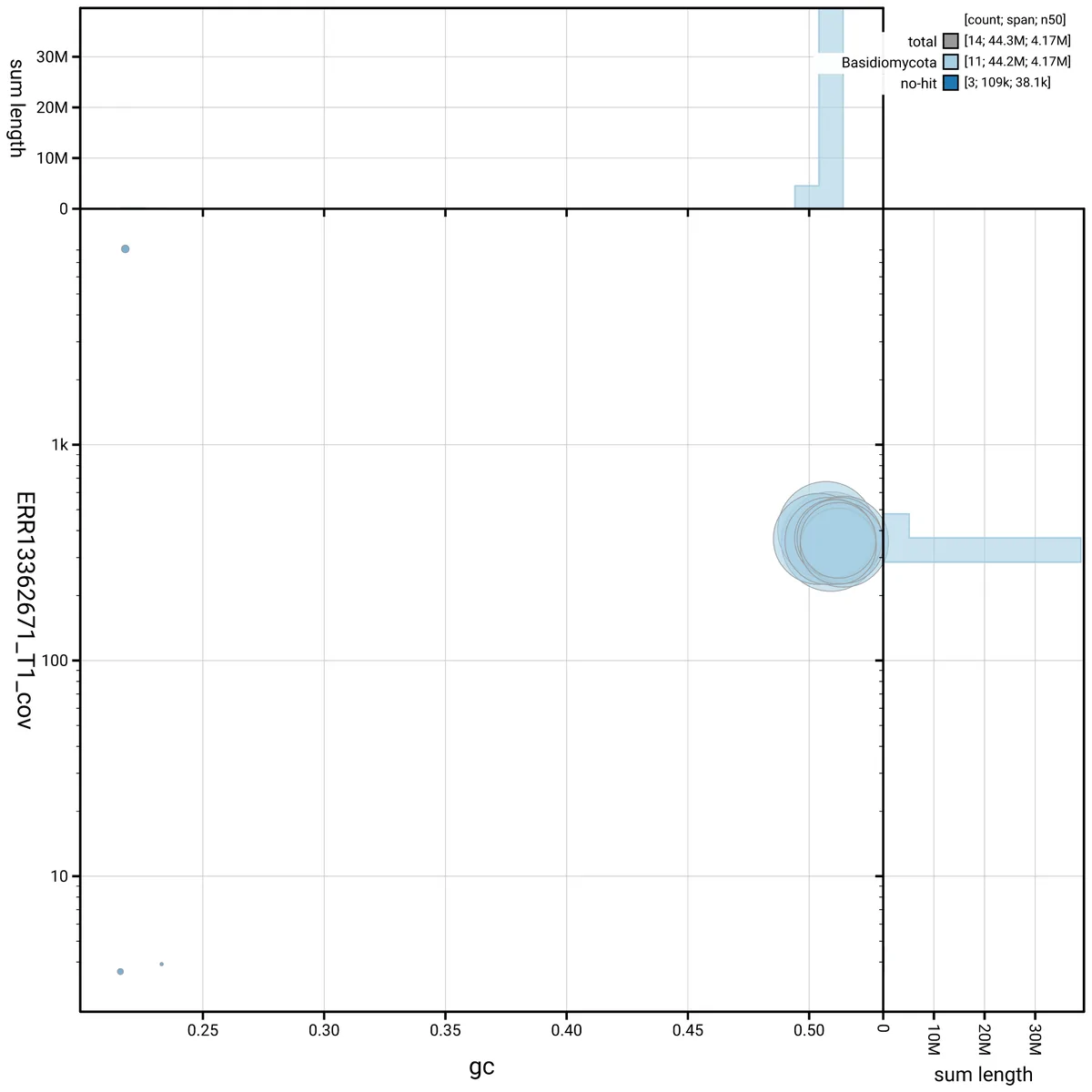
BlobToolKit blob plot for gfRusNigr1.hap1.1. The plot shows base coverage (vertical axis) and GC content (horizontal axis). The circles represent scaffolds, with the size proportional to scaffold length and the colour representing phylum membership. The histograms along the axes display the total length of sequences distributed across different levels of coverage and GC content. An interactive version of this figure is available on the BlobToolKit viewer.

Table 4 lists the assembly metric benchmarks adapted from Rhie *et al.*. (2021) and the Earth BioGenome Project (EBP) Report on Assembly Standards. The three-part EBP metric calculated for haplotype 1 is **6.C.Q64**, meeting the 6.C.Q40 benchmark.

**Table 4.** Earth BioGenome Project summary metrics for the *R. nigricans* assembly

**Measure**	**Value**	**Benchmark**
EBP summary (haplotype 1)	6.C.Q64	6.C.Q40
Contig N50 length	3.31 Mb	> 1 Mb
Scaffold N50 length	4.17 Mb	= chromosome N50
Consensus quality (QV)	Haplotype 1: 64.7; haplotype 2: 65.7; combined: 65.2	≥ 40
*k*-mer completeness	Haplotype 1: 83.88%; Haplotype 2: 83.96%; combined: 99.43%	> 90%
BUSCO	Haplotype 1: C:92.8% [S:92.3%, D:0.5%], F:1.2%, M:6.0%, n:2,898; Haplotype 2: C:92.8% [S:92.2%, D:0.5%], F:1.3%, M:5.9%, n:2,898	*S > 90%*
Percentage of assembly assigned to chromosomes	99.88%	≥ 90%

**Notes:** The EBP summary uses log10(Contig N50); chromosome-level (C) or log10(Scaffold N50); Q (Merqury QV). BUSCO v5.5.0 using the agaricomycetes_odb10 lineage dataset: C=complete; S=single-copy; D=duplicated; F=fragmented; M=missing; n=orthologues.

### Genome annotation report

The *R. nigricans* genome assembly (GCA_964656295.1) was annotated by Ensembl at the European Bioinformatics Institute (EBI). This annotation includes 22,854 transcribed mRNAs from 13,918 protein-coding and 2,119 non-coding genes. The average transcript length is 1,957.02 bp, with an average of 1.43 coding transcripts per gene and 5.36 exons per transcript. For further information about the annotation, please refer to the annotation page on Ensembl.

### Data availability

European Nucleotide Archive: Russula nigricans. Accession number PRJEB77549; https://identifiers.org/ena.embl/PRJEB77549. The genome sequence is released openly for reuse. The *R. nigricans* genome sequencing initiative is part of the Darwin Tree of Life Project ( PRJEB40665) and the Sanger Institute Tree of Life Programme ( PRJEB43745). All raw sequence data and the assembly have been deposited in INSDC databases. Raw data and assembly accession identifiers are reported in Table 1 and Table 2.

Pipelines used for genome assembly at the WSI Tree of Life are available at https://pipelines.tol.sanger.ac.uk/pipelines. Table 5 lists software versions used in this study.

**Table 5.** Software versions and sources used for *R. nigricans*

**Software**	**Version**	**Source**
BLAST	2.14.0	https://ftp.ncbi.nlm.nih.gov/blast/executables/blast+/
BlobToolKit	4.3.9	https://github.com/genomehubs/blobtoolkit
blobtk	0.5.1	https://github.com/genomehubs/blobtk
BUSCO	5.5.0; 6.1.0	https://gitlab.com/ezlab/busco
bwa-mem2	2.2.1	https://github.com/bwa-mem2/bwa-mem2
DIAMOND	2.1.8	https://github.com/bbuchfink/diamond
fasta_windows	0.2.4	https://github.com/tolkit/fasta_windows
FastK	1.1	https://github.com/thegenemyers/FASTK
GenomeScope	2.1.0	https://github.com/tbenavi1/genomescope2.0
Gfastats	1.3.6	https://github.com/vgl-hub/gfastats
Hifiasm	0.19.8-r603	https://github.com/chhylp123/hifiasm
HiGlass	1.13.4	https://github.com/higlass/higlass
MerquryFK	1.1.0-c1	https://github.com/thegenemyers/MERQURY.FK
Minimap2	2.24-r1122	https://github.com/lh3/minimap2
MitoHiFi	3.2.2	https://github.com/marcelauliano/MitoHiFi
Oatk	1.0	https://github.com/c-zhou/oatk
MultiQC	1.14; 1.17 and 1.18	https://github.com/MultiQC/MultiQC
Nextflow	23.10.0	https://github.com/nextflow-io/nextflow
PretextSnapshot	0.0.6	https://github.com/sanger-tol/PretextSnapshot
PretextView	1.0.3	https://github.com/sanger-tol/PretextView
samtools	1.19.2	https://github.com/samtools/samtools
sanger-tol/ascc	0.1.0	https://github.com/sanger-tol/ascc
sanger-tol/blobtoolkit	0.6.0	https://github.com/sanger-tol/blobtoolkit
sanger-tol/curationpretext	1.4.2	https://github.com/sanger-tol/curationpretext
Seqtk	1.4-r122	https://github.com/lh3/seqtk
Singularity	3.9.0	https://github.com/sylabs/singularity
TreeVal	1.1.1	https://github.com/sanger-tol/treeval
YaHS	1.2a.2	https://github.com/c-zhou/yahs

### Author information

- Members of the Royal Botanic Gardens Kew Genome Acquisition Lab
- Members of the Darwin Tree of Life Barcoding collective
- Members of the Wellcome Sanger Institute Tree of Life Management, Samples and Laboratory team
- Members of Wellcome Sanger Institute Scientific Operations – Sequencing Operations
- Members of the Wellcome Sanger Institute Tree of Life Core Informatics team
- Members of the Tree of Life Core Informatics collective
- Members of the Darwin Tree of Life Consortium

### Wellcome Sanger Institute – Legal and Governance

The materials that have contributed to this genome note have been supplied by a Darwin Tree of Life Partner. The submission of materials by a Darwin Tree of Life Partner is subject to the **‘Darwin Tree of Life Project Sampling Code of Practice’**, which can be found in full on the Darwin Tree of Life website. By agreeing with and signing up to the Sampling Code of Practice, the Darwin Tree of Life Partner agrees they will meet the legal and ethical requirements and standards set out within this document in respect of all samples acquired for, and supplied to, the Darwin Tree of Life Project. Further, the Wellcome Sanger Institute employs a process whereby due diligence is carried out proportionate to the nature of the materials themselves, and the circumstances under which they have been/are to be collected and provided for use. The purpose of this is to address and mitigate any potential legal and/or ethical implications of receipt and use of the materials as part of the research project, and to ensure that in doing so we align with best practice wherever possible. The overarching areas of consideration are:

- Ethical review of provenance and sourcing of the material
- Legality of collection, transfer and use (national and international)

Each transfer of samples is further undertaken according to a Research Collaboration Agreement or Material Transfer Agreement entered into by the Darwin Tree of Life Partner, Genome Research Limited (operating as the Wellcome Sanger Institute), and in some circumstances, other Darwin Tree of Life collaborators.
