# Supplementary material for: The genome sequence of Russula nigricans Fr., 1838 (Russulales: Russulaceae)
Source: Wellcome Open Res. 2026 Oct 9;11:846. [Version 1] doi: 10.12688/wellcomeopenres.28091.1 (PMC13680837; doi:10.12688/wellcomeopenres.28091.1)
Supplement: Peer review history

# Reviewer report: Reviewer response for version 1

**DOI:** 10.21956/wellcomeopenres.30907.r173525

**Published:** 2026-10-19

**Authors:** Brenda Wingfield

**Affiliations:** 1 University of Pretoria, Pretoria, South Africa

This article describes the generation of a high-quality reference genome assembly for *Russula nigricans* (Blackening Brittlegill), produced as part of the Darwin Tree of Life project. In addition to sequencing the nuclear genome, the authors provide phased assemblies representing two haplotypes, with total lengths of 44.21 Mb and 45.01 Mb, respectively, each comprising 11 chromosomal pseudomolecules and thus providing an exceptionally complete representation of the genome. Appropriate bioinformatic tools were used during assembly and curation to identify and remove potential contamination, supporting the quality of the final genome resource. The mitochondrial genome was also assembled and is 54.23 kb in length, while annotation of the nuclear assembly identified 13,918 protein-coding genes. Importantly, the underlying sequence and assembly data are readily accessible, providing a valuable genomic resource for future comparative, evolutionary, population-genomic and functional studies of *R. nigricans *and related Russulaceae.

Are the datasets clearly presented in a usable and accessible format, and the assembly and annotation available in an appropriate subject-specific repository?

Yes

Are sufficient details of the sequencing and extraction, software used, and materials provided to allow replication by others?

Yes

Are the rationale for sequencing the genome and the species significance clearly described?

Yes

Are the protocols appropriate and is the work technically sound?

Yes

Reviewer Expertise:

Fungal genomics

I confirm that I have read this submission and believe that I have an appropriate level of expertise to confirm that it is of an acceptable scientific standard.

# Reviewer report: Reviewer response for version 1

**Published:** 2026-10-09

**Authors:** Automated Benchmarking

**Affiliations:** 1 Wellcome Sanger Institute, Hinxton, UK

As this article is a Genome Note, Automated Benchmarking has been used to determine whether the reported assembly passes four defined checks established by the Earth BioGenome Project: the raw and assembled data are publicly available, the Earth BioGenome Project three-part metric is met, the k-mer completeness exceeds 90%, and the BUSCO score passes the stated threshold. For Russula nigricans (gfRusNigr1), all four checks passed. Data available: pass. ENA study PRJEB77549, raw-read accessions ERR13362671, ERR13363453, ERR13363452, and ERR15140896, and assembly accessions GCA_964656295.1 and GCA_964656355.1 are public. Earth BioGenome Project three-part metric: pass. Haplotype 1 is reported as 6.C.Q64, meeting the 6.C.Q40 reference standard. k-mer completeness: pass. The combined haplotype assemblies have 99.43% k-mer completeness, exceeding the Earth BioGenome Project target of 90%. BUSCO: pass. Haplotype 1, which is proposed as the reference assembly for this species, has 92.8% complete BUSCOs (92.3% single-copy and 0.5% duplicated). BUSCO software version: 5.5.0; lineage dataset: agaricomycetes_odb10. It meets the target of greater than 90% complete and single-copy BUSCOs. This decision uses values reported in the Genome Note and public accession records.

Reviewer Expertise:

NA

I confirm that I have read this submission and believe that I have an appropriate level of expertise to confirm that it is of an acceptable scientific standard.
